# Activities of artesunate-based combinations and tafenoquine against *Babesia bovis in vitro* and *Babesia microti in vivo*

**DOI:** 10.1186/s13071-020-04235-7

**Published:** 2020-07-20

**Authors:** Leonardo J. M. Carvalho, Bunduurem Tuvshintulga, Arifin B. Nugraha, Thillaiampalam Sivakumar, Naoaki Yokoyama

**Affiliations:** 1 National Research Center for Protozoan Diseases, Obihiro University of Agriculture and Veterinary Medicine, Hokkaido, Japan; 2 Research Center for Global Agromedicine, Obihiro University of Agriculture and Veterinary Medicine, Hokkaido, Japan; 3 Laboratory of Malaria Research, Oswaldo Cruz Institute, Fiocruz, Rio de Janeiro, Brazil; 4 OIE Reference Laboratory for Bovine Babesiosis and Equine Piroplasmosis, National Research Center for Protozoan Diseases, Obihiro University of Agriculture and Veterinary Medicine, Hokkaido, Japan

**Keywords:** Babesia, Artesunate, Tafenoquine

## Abstract

**Background:**

Babesiosis represents a veterinary and medical threat, with a need for novel drugs. Artemisinin-based combination therapies (ACT) have been successfully implemented for malaria, a human disease caused by related parasites, *Plasmodium* spp. The aim of this study was to investigate whether ACT is active against *Babesia in vitro* and *in vivo*.

**Methods:**

Mefloquine, tafenoquine, primaquine, methylene blue and lumefantrine, alone or in combination with artesunate, were tested *in vitro* against *Babesia bovis*. Parasite growth was verified using a SYBR green I-based fluorescence assay. Mice infected with *Babesia microti* were treated with mefloquine or tafenoquine, alone or in combination with artesunate, and parasitemia was verified by microscopy and PCR.

**Results:**

All drugs, except lumefantrine, showed *in vitro* activity against *B*. *bovis*, with methylene blue showing the most potent activity (concentration 0.2 μM). Combination with artesunate led to improved activity, with mefloquine showing a striking 20-fold increase in activity. Tafenoquine (10 mg/kg, base), combined or not with artesunate, but not mefloquine, induced rapid clearance of *B*. *microti in vivo* by microscopy, but mice remained PCR-positive. Blood from mice treated with tafenoquine alone, but not with tafenoquine-artesunate, was infective for naive mice upon sub-inoculation.

**Conclusions:**

Tafenoquine, and most likely other 8-aminoquinoline compounds, are promising compounds for the development of ACT for babesiosis.
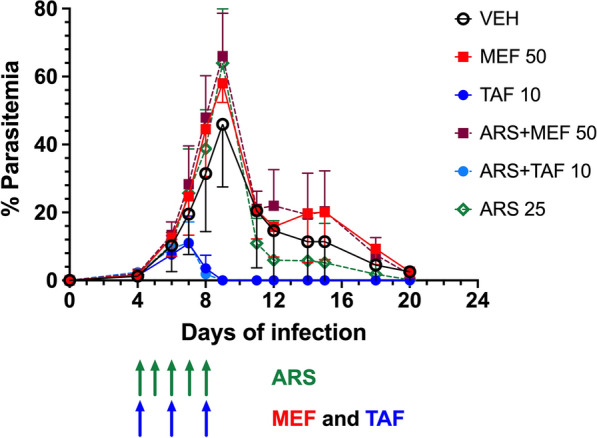

## Background

The genus *Babesia* contains a very diverse group of piroplasmid organisms, such as *Babesia bovis*, *Babesia bigemina*, *Babesia microti* and *Babesia caballi* [1]. Babesiosis caused by these *Babesia* species represents a veterinary and medical threat. In the case of animal babesiosis, diminazene aceturate and imidocarb have been established as effective therapeutic agents [2]. However, the side effects associated with the administration of these drugs and reports of resistance indicate the need for the discovery and development of new drugs for treating animal babesiosis. Human babesiosis is caused largely by a rodent parasite *B*. *microti* and can lead to severe and even fatal infections especially in immunocompromised patients [3]. However, the currently established treatments, the combinations of quinine plus clindamycin or atovaquone plus azithromycin, are limited because of substantial adverse effects and lack of efficacy due to drug resistance [4–6]. Therefore, there is an active search for new drugs to treat babesiosis.

A number of compounds have been screened in the past years, some of them showing promising results. Among the classes of compounds that have been screened, antimalarial drugs are prominent [7], because *Plasmodium* spp. and *Babesia* spp. are closely related organisms, often sharing same drug targets. As mentioned, the antimalarial drugs quinine and atovaquone are currently used to treat human babesiosis, and radical cure has been demonstrated in experimental *B*. *microti* infection using a novel atovaquone-based drug combination with an endochin-like quinolone [8]. Atovaquone has also been shown to be highly active against *B*. *bovis* and *B*. *divergens* [9]. Methylene blue, a ‘rediscovered’ antimalarial drug, has been shown to be active *in vitro* against *Babesia* and *Theileria* parasites, but the activity *in vivo* was disappointing [10]. Out of several compounds with antimalarial activity, including mefloquine, halofantrine, artesunate, artelenic acid and the combination quinine plus clindamycin, only two 8-aminoquinolines, WR006026 and WR238605 (“tafenoquine”), were able to cause a 100% suppression of *B. microti* infection in the hamster model [11]. The 8-aminoquinolines, indeed, show consistent activity against *Babesia*. A recent study confirmed the potent activity of tafenoquine against *B. microti* infection [12], and similar results have been obtained with primaquine and 4-methyl-primaquine [13, 14]. More recently, extensive screening of the malaria box compound library led to the identification of novel leads with promising antibabesial activity *in vitro* and *in vivo* [7, 9, 15]. Artemisinin derivatives have shown substantial inhibitory activity against different species of *Babesia in vitro*, but only limited activity *in vivo* [11, 16–21]. In the case of malaria, one successful strategy to improve treatment efficacy has been the use of drug combination therapies, that is, the use of two or more drugs with different mechanisms of action to overcome low efficacy, resistance and pharmacokinetic limitations. Today, the use of the so-called ACTs (artemisinin combination therapies) has become the first line of treatment against non-complicated malaria in most endemic countries, in two- or three-drug combinations [22]. This strategy has not been properly explored in babesiosis. One of the few studies indicated that the combination of artemisinin and lumefantrine *in vitro* had a synergistic effect against *B. gibsoni* [20]. The activity and the efficacy of different artemisinin-based combinations have not been explored in greater detail with other *Babesia* species of veterinary and medical importance, such as *B*. *bovis* and *B*. *microti*. Therefore, we propose to address the suitability of different ACTs as potential antibabesial drugs in *in vitro* and *in vivo* studies, using established drug partners of artemisinins (lumefantrine and mefloquine) as well as new potential combinations (primaquine, tafenoquine and methylene blue).

## Methods

### Aim, design and setting of the study

This study was designed to investigate the activity of selected antimalarial drugs, alone or in combination with artesunate, against *B*. *bovis in vitro* and the efficacy of the most active combinations *in vivo* against *B*. *microti*. For the *in vitro* studies, each drug was added to *B*. *bovis* cultures at serial dilutions, alone or in combination with artesunate. Drugs that showed activity *in vitro* alone and with an improved profile when combined with artesunate were selected for *in vivo* efficacy testing using BALB/c mice infected with *B*. *microti*. All studies were performed at the National Research Center for Protozoan Diseases, Obihiro, Japan.

### *In vitro Babesia* growth inhibition assay

*Babesia bovis* (Texas strain) was cultivated in purified bovine red blood cells (RBCs) using a microaerophilic, stationary-phase culture system, as previously described [23]. *Babesia bovis*-infected RBCs (iRBCs) were cultivated at 1% parasitemia and 2.5% hematocrit in 96-well plates using M199 media containing 40% bovine serum with or without the following drugs in serial dilutions: lumefantrine (LUM: 0.1–200 μM), mefloquine hydrochloride (MEF: 0.1–100 μM), primaquine bisphosphate (PRI: 0.1–100 μM), tafenoquine succinate (TAF: 0.1–100 μM), and methylene blue (MB: 0.01–1 μM), alone or in combination with sodium artesunate (ARS: 0.1–100 μM) (all drugs were purchased from Sigma-Aldrich, Tokyo, Japan). Stock solutions for all drugs were prepared using DMSO (Sigma-Aldrich) as a solvent, except for methylene blue (MilliQ water as solvent). The cultures, in triplicate wells for each concentration of the drugs, were incubated in an atmosphere of 5% O_2_ and 5% CO_2_ at 37 °C for 4 days without replacement of the medium. Maximum final concentration of DMSO in the wells ranged from 0.1% to 0.5%, and control wells (no drug) were prepared using culture medium containing DMSO at these concentrations. Assays were run in triplicate for each drug, and at least three independent assays were run for each drug. At the end of the 4-day incubation period, parasite growth was measured using a fluorescent-based assay, as previously described [24, 25]. Briefly, SYBR green (Lonza Rockland Inc., Rockland, ME, USA) diluted 1:10,000 in lysing buffer was added to the wells and incubated at room temperature in the dark for 4 h. Then, plates were read in a fluorescence spectrophotometer (Fluoroskan Ascent; Thermo Fisher Scientific, Waltham, MA, USA) (emission wavelength: 450nm; absorbance wavelength: 518 nm) and the half maximal inhibitory concentrations (IC_50_s) were calculated [26].

### *In vivo* tests: chemotherapeutic evaluation in mice

All protocols were approved by the Obihiro University ethical committee on animal experimentation (approval number: 19-105). BALB/c female mice (8–9-weeks-old, CLEA, Tokyo, Japan), kept under specific pathogen free conditions and divided equally into groups of 5 mice each, were inoculated intraperitoneally (IP) with 1 × 10^7^*B*. *microti* (Munich strain) iRBCs following a method described by Igarashi et al. [27]. ARS, MEF and TAF were dissolved in 5% sodium bicarbonate solution, 20% ethanol/80% PEG 400 50% in autoclaved double distilled water (DDW), and 10% DMSO/90% PEG 400 50% in autoclaved DDW, respectively. Five percent sodium bicarbonate and 10% DMSO/90% PEG 400 50% in autoclaved DDW were used as vehicle control. On day 4 of the infection, mice received the first dose of each treatment: vehicle (VEH) PO or IP ARS 25 mg/kg IP (100 μl); MEF 50 mg/kg PO (200 μl); TAF 10 mg/kg PO (200 μl); ARS IP + MEF PO; and ARS IP + TAF PO. ARS was given daily for 5 days, whereas MEF or TAF were given in alternate days (4, 6, and 8 days post-infection). The vehicle group received both 5% sodium bicarbonate IP (100 μl) daily and 10% DMSO/90% PEG400 50% PO (200 μl) in alternate days (days 4, 6, and 8 post-infection). Parasitemia was checked daily by microscopy using Giemsa-stained smears (and also by PCR on days 15, 20 and 27 post-infection) from blood taken from the tip of the tail. Body weight was checked every 2–4 days and blood parameters (hematocrit, platelet counts, etc.) were measured on days 4, 8, 12, 15 and 20 post-infection. Mice were followed up to 30 days and then subjected to euthanasia (pre-anesthesia with isoflurane 4.5% for induction followed by cervical dislocation).

### Parasite detection by PCR

PCR was undertaken to detect parasite DNA [28] in blood of *B. microti*-infected, TAF-treated and ARS-TAF-treated mice on days 15, 20 and 27 post-infection. DNA was prepared from the blood of these mice and the non-treated control group using the boiling method [29]. Briefly, 10 µl blood from each mouse was incubated at 100 °C for 5 min and centrifuged at 10,000× *rpm*, and then the supernatant containing DNA was collected. PCR targeting the *B. microti* small subunit rRNA (*SSU* rRNA) gene was carried out as described previously [30], using the following nested PCR primer sets: outer forward primer Babl (5’-CTT AGT ATA AGC TTT TAT ACA GC-3’)/outer reverse primer Bab4 (5’-ATA GGT CAG AAA CTT GAA TGA TAC A-3’) and inner forward primer Bab2 (5’-GTT ATA GTT TAT TTG ATG TTC GTT T-3’)/inner reverse primer Bab3 (5’-AAG CCA TGC GAT TCG CTA AT-3’). The expected size of the final PCR product was 154 bp.

### Parasite sub-inoculation

In the TAF-treated and ARS-TAF-treated groups, 30 days after inoculation (22 days after last treatment dose), 10 μl of blood was taken from each of the 5 mice, pooled (50 μl total), diluted to a final volume of 500 μl in sterile saline and inoculated IP in one mouse per group. Parasitemia was then checked daily by microscopy for up to 30 days.

### Statistical analysis

Differences in parasitemia, body weight, hematocrit and platelet counts between untreated (vehicle) and treated groups at each timepoint were analyzed by Student’s t-test for independent samples using GraphPad Prism software (GraphPad, San Diego, CA, USA), and *P* values of < 0.05 were considered statistically significant.

## Results

### *In vitro* susceptibility of *B. bovis* to antimalarial drugs

**Fig. 1:**
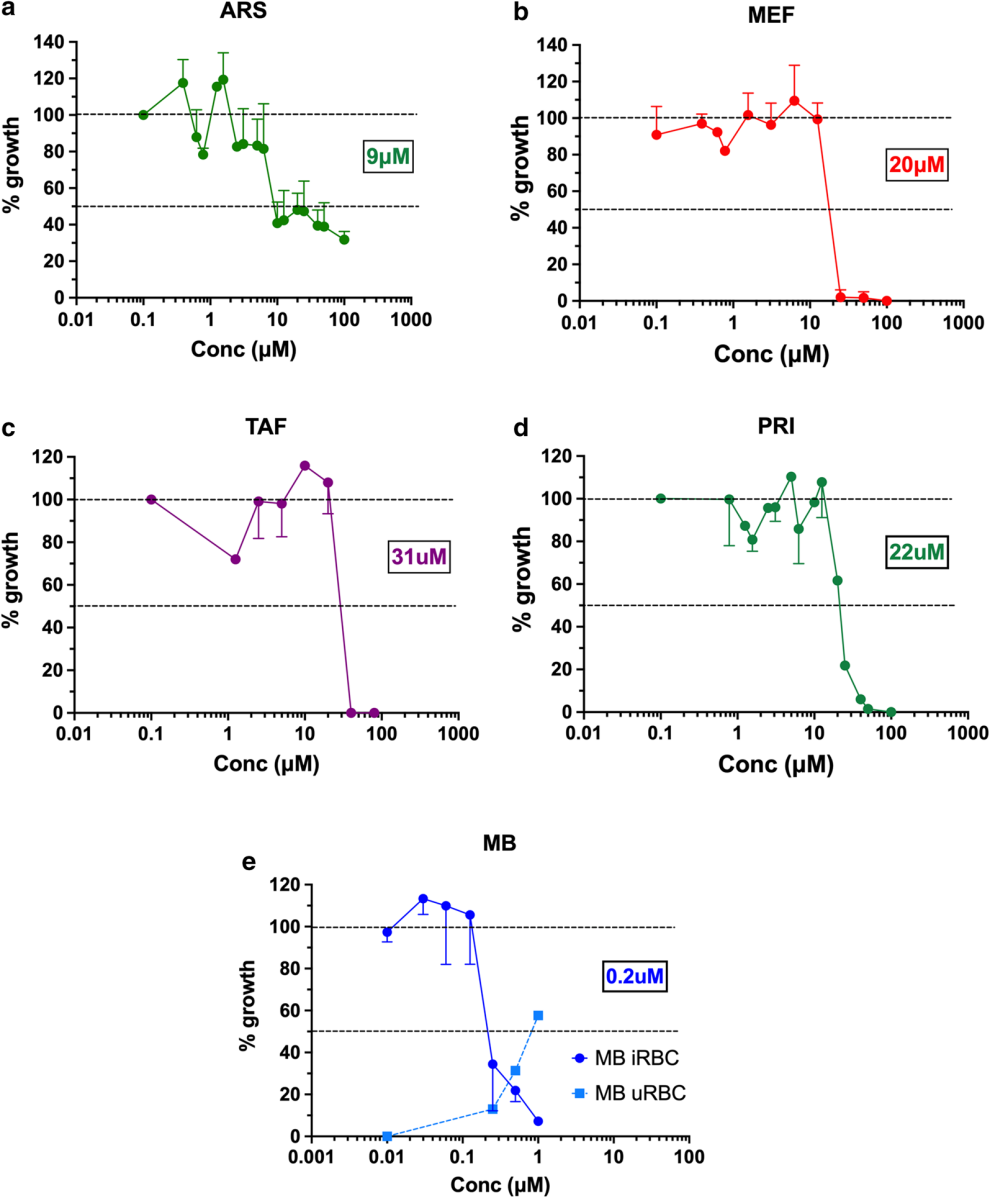
*In vitro* activity of artesunate (ARS) (**a**), mefloquine (MEF) (**b**), tafenoquine (TAF) (**c**), primaquine (PRI) (**d**) and methylene blue (MB) (**e**) against *Babesia bovis*. Results are expressed as % (mean ± standard deviation) of growth at each drug concentration in relation to wells without drug (100% growth) after 4 days of culture. In the MB graph (**e**), wells containing uninfected red blood cells (uRBC) were run in parallel for all MB concentrations, because MB causes a background fluorescence and this background value was subtracted from the corresponding wells containing infected red blood cells (iRBC) to generate the iRBC MB drug response curve. Numbers in boxes in each graph represent the IC_50_ (in µm) for each given drug. *Abbreviation*: Conc (x-axis), concentration of the drug

First, the activity of each individual drug was assessed *in vitro* against *B*. *bovis*. Five of the six antimalarial drugs assayed had activity against *B*. *bovis* (Fig. 1), with the exception of lumefantrine, which showed no activity up to 200 μM (data not shown). The most active drug was MB, with an IC_50_ of 0.2 μM. The other drugs showed activity above 1 μM, varying between 9 μM (ARS) and 31 μM (TAF). While MEF, TAF, PRI and MB were able to kill all parasites at higher concentrations (above 20 μM), ARS was unable to eliminate all parasites even at the concentration of 100 μM.

**Fig. 2:**
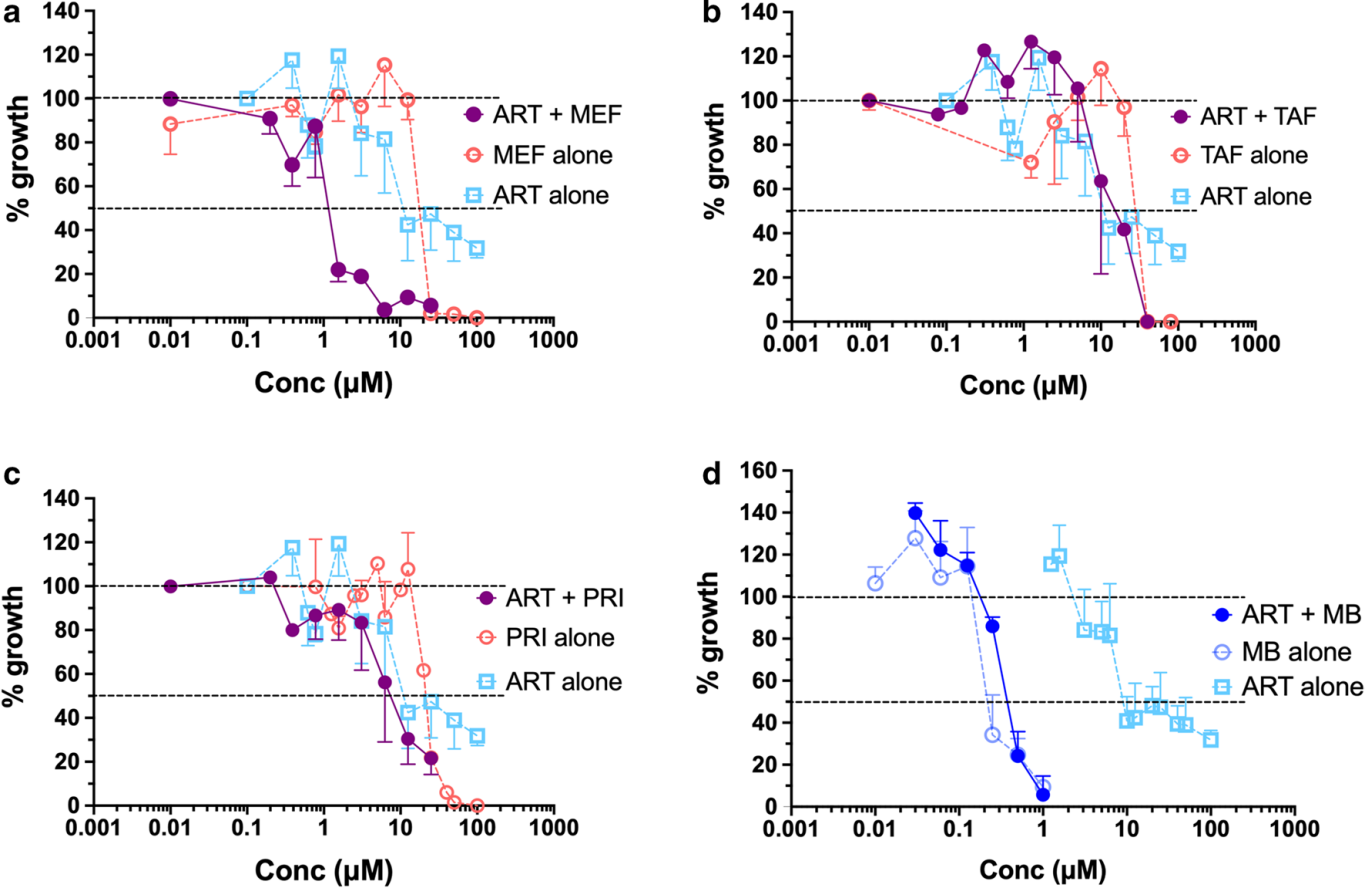
*In vitro* activity of mefloquine (MEF) (**a**), tafenoquine (TAF) (**b**), primaquine (PRI) (**c**) and methylene blue (MB) (**d**), alone or in combination with artesunate (ARS), against *Babesia bovis*. Drugs were combined in equimolar concentrations, except for methylene blue where the proportion for each well was 25:1 ARS:MB. Results are expressed as % of growth (mean ± standard deviation) at each drug concentration in relation to wells without drug (100% growth) after 4 days of culture. *Abbreviation*: Conc (x-axis), concentration of the drug

Next, combinations of artesunate with each of the four drugs were assessed to verify whether it resulted in better activity than each drug alone against *B*. *bovis*. This was shown to be the case for MEF, TAF and PRI, but not MB (Fig. 2). The most potent combination effect was observed for ARS-MEF; MEF alone showed an IC_50_ of 20 μM, whereas the ARS-MEF combination showed an IC_50_ of 1 μM (20 times more potent). The combinations ARS-TAF (15 μM) and ARS-PRI (7 μM) were shown to be about 2–3 times more potent than TAF (31 μM) or PRI (22 μM) alone. In both cases, the combination did not result in an IC_50_ substantially better than that of ARS alone (9 μM), but improved killing efficacy at higher concentrations. In the case of MB, the IC_50_ of the combination was 0.4 μM, higher than that of MB alone (0.2 μM).

### *In vivo* susceptibility of *B. microti* to antimalarial drugs

**Fig. 3:**
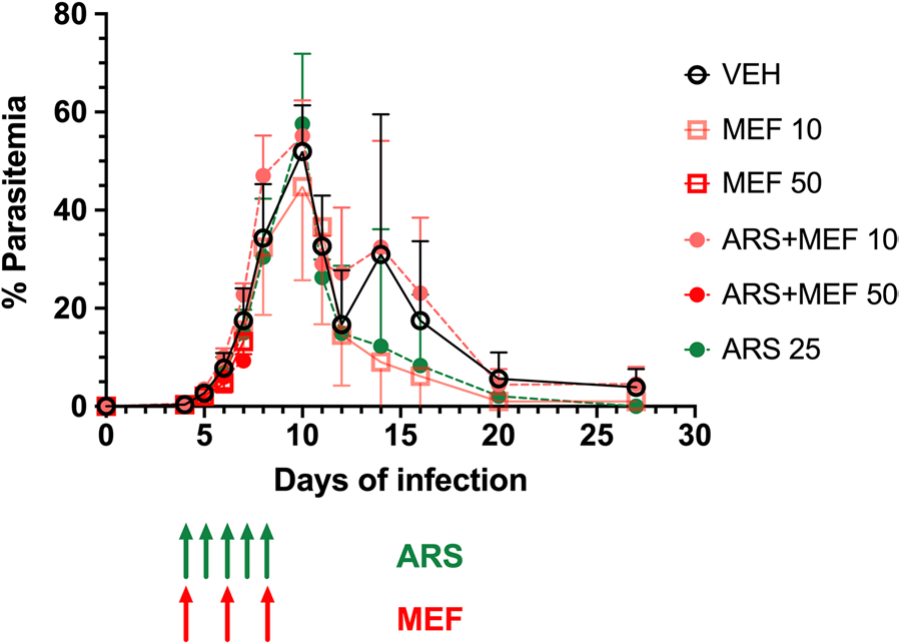
Course of parasitemia (mean ± standard deviation) in BALB/c mice inoculated with 1 × 10^7^*Babesia microti* and treated with either vehicle, artesunate (ARS) 25 mg/kg, mefloquine (MEF) 10 mg/kg or 50 mg/kg, artesunate (ARS) 25 mg/kg + mefloquine (MEF) 10 mg/kg or 50 mg/kg (5 mice per group). Drugs were dissolved in 5% sodium bicarbonate (ARS) or 20% ethanol in polyethylene glycol in water (MEF) and administered intraperitoneally. ARS was given daily for 5 days starting at day 4 of infection (green arrows) and MEF was given in alternate days (days 4, 6 and 8: red arrows). Parasitemia was determined by microscopy. No significant differences in the course of parasitemia were observed between the groups

**Fig. 4:**
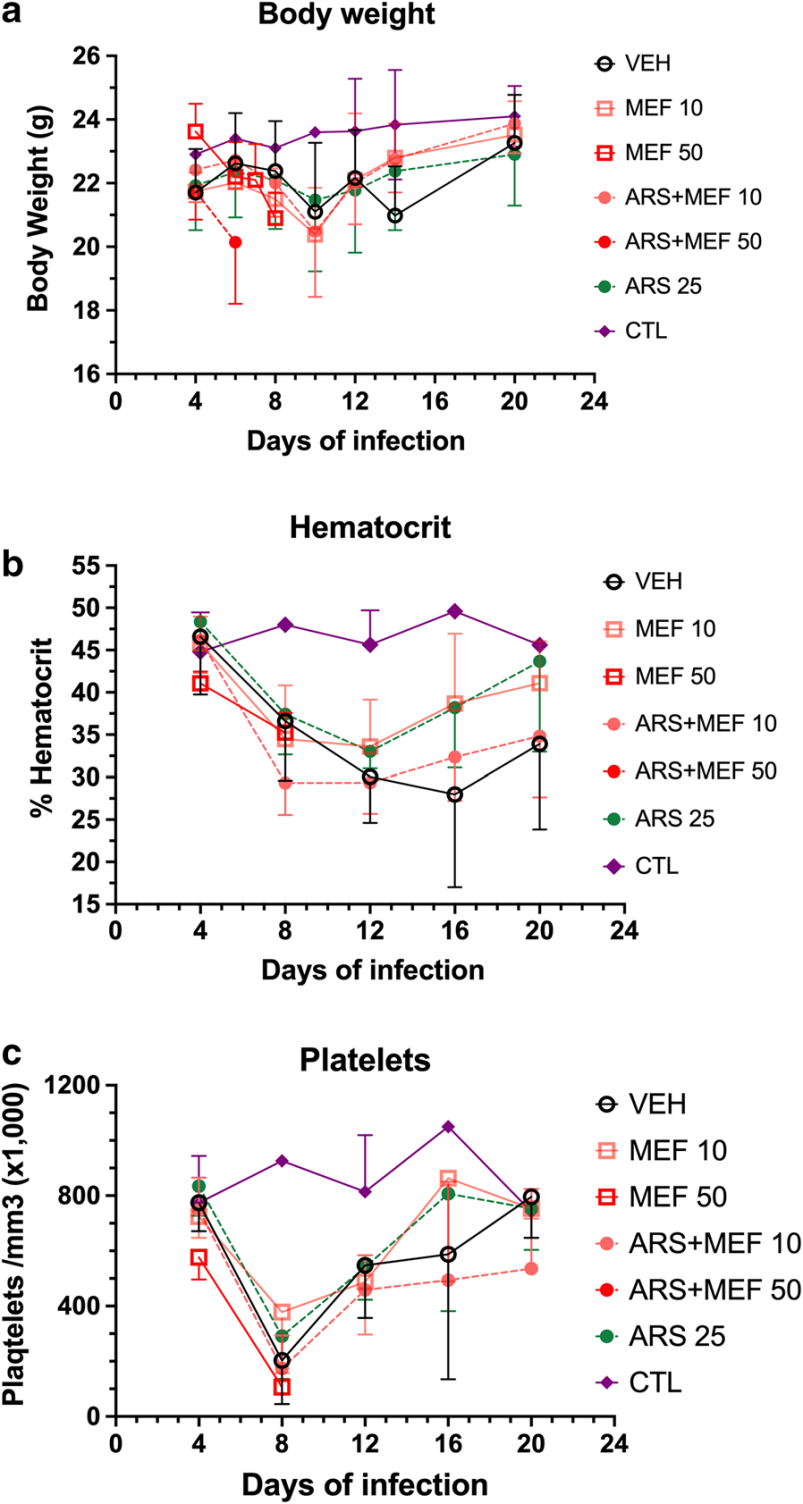
Body weight (**a**), hematocrit (**b**) and platelet counts (**c**) (mean ± standard deviation) of the uninfected control (CTL) and BALB/c mice inoculated with 1 × 10^7^*Babesia microti* and treated with either vehicle, artesunate (ARS) 25 mg/kg, mefloquine (MEF) 10 mg/kg or 50 mg/kg, artesunate (ARS) 25 mg/kg + mefloquine (MEF) 10 mg/kg or 50 mg/kg (5 mice per group). **a** Infected mice receiving mefloquine 50 mg/kg in both groups showed discomfort after drug administration and were subjected to euthanasia by day 8. Infected mice in the other groups showed transient loss of weight at day 10, around the peak parasitemia, and then recovered, and there were no significant differences in body weight changes between the groups. Infected mice of all groups showed marked decreases in hematocrit (**b**) and platelet counts (**c**), but there were no significant differences between the groups

In view of the substantial improvement in the IC_50_ of the combination ARS-MEF (1 μM) compared to MEF alone (20 μM), this drug combination was selected for *in vivo* testing against *B*. *microti*. The ARS dose was fixed at 25 mg/kg (5 daily doses, starting at day 4 of infection) and 2 different doses of MEF were defined: 10 mg/kg and 50 mg/kg (3 injections in alternate days: 4, 6 and 8 days post-infection). For this experiment, the intraperitoneal (IP) route was chosen. However, either each drug alone or the combinations did not inhibit parasite growth (Fig. 3). MEF at 50 mg/kg IP was shown to be toxic, with animals showing signs of discomfort after drug administration, and therefore treatment was discontinued, and mice were subjected to euthanasia. In all other groups, infected mice showed slight body weight loss around the parasitemia peak but quickly recovered, with no differences in body weight changes between the groups. Marked decreases in hematocrit and platelet counts were observed in all groups during infection, but with no differences in the magnitude between groups (Fig. 4).

**Fig. 5:**
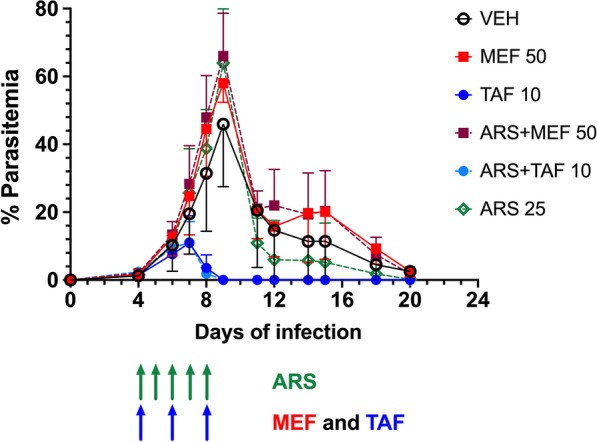
Course of parasitemia (mean ± standard deviation) in BALB/c mice inoculated with 1 × 10^7^*Babesia microti* and treated with either vehicle, artesunate (ARS) 25 mg/kg, mefloquine (MEF) 50 mg/kg, tafenoquine (TAF) 10 mg/kg (base), artesunate (ARS) 25 mg/kg + mefloquine (MEF) 50 mg/kg and artesunate (ARS) 25 mg/kg + tafenoquine (TAF) 10 mg/kg (base). ARS dissolved in 5% sodium bicarbonate was administered intraperitoneally, MEF dissolved in 20% ethanol in polyethylene glycol in water was given orally, and TAF dissolved in 10% DMSO in polyethylene glycol in water was given orally. ARS was given daily for 5 days starting at day 4 of infection (green arrows), and MEF or TAF were given in alternate days (days 4, 6 and 8: blue arrows). Parasitemia was checked by microscopy. Mice in both TAF groups showed lower levels of parasitemia on day 8 (*P* = 0.0079 for all groups compared with each TAF group) and all mice cleared parasitemia on day 9. The parasitemia of the mice in the other groups (ARS alone or MEF) were not significantly different from the parasitemia in the vehicle group

**Fig. 6:**
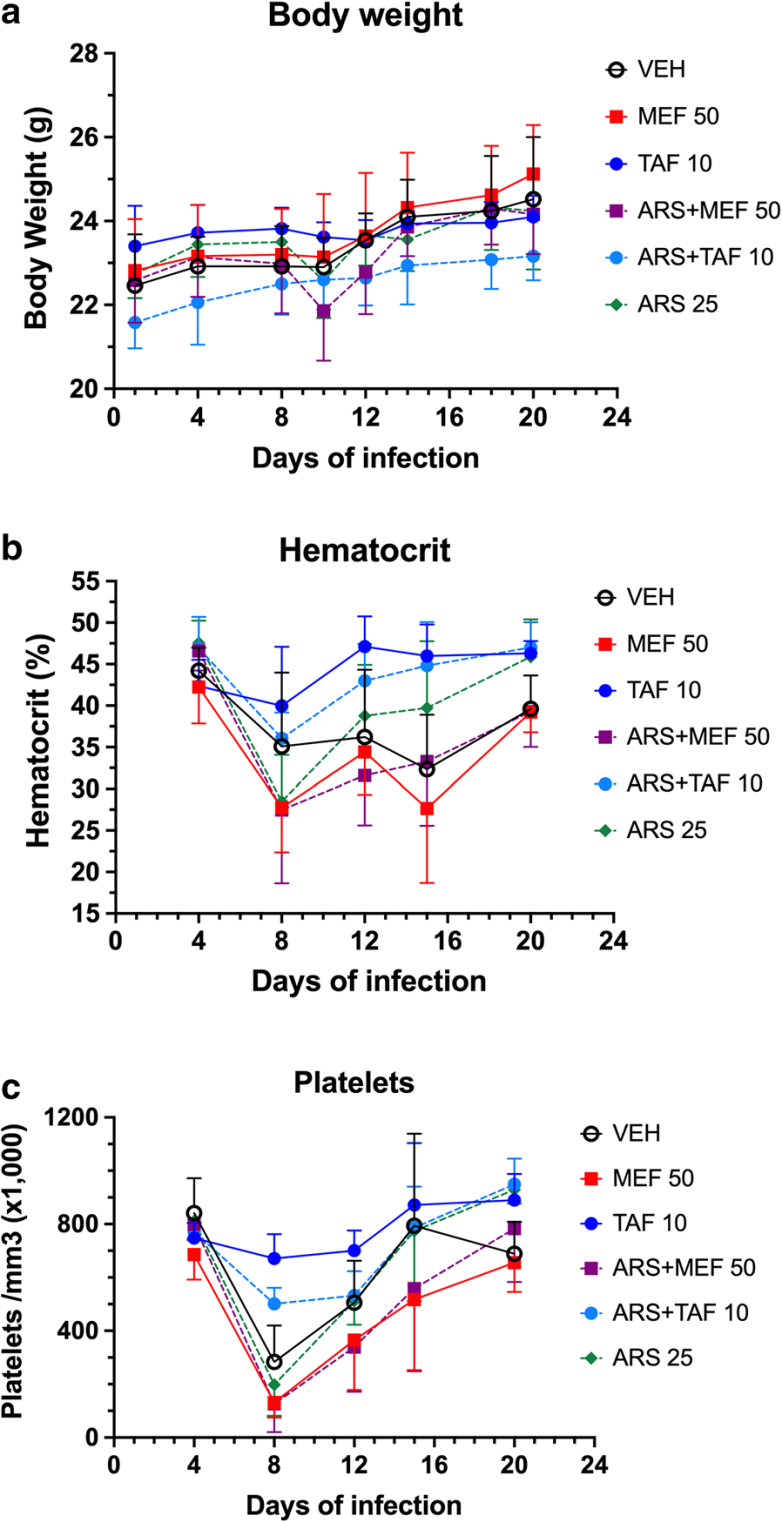
Body weight (**a**), hematocrit (**b**) and platelet counts (**c**) (mean ± standard deviation) of BALB/c mice inoculated with 1 × 10^7^*Babesia microti* and treated with either vehicle, artesunate (ARS) 25 mg/kg, mefloquine (MEF) 50 mg/kg, tafenoquine (TAF) 10 mg/kg (base), ARS 25 mg/kg + MEF 50 mg/kg and ARS 25 mg/kg + TAF 10 mg/kg (base) (5 mice per group). **a** There was no significant difference in body weight changes between groups during infection, and mice in all groups showed a 3–10% increase of weight at day 20. **b** Hematocrit decreased in all groups and on days 8 and 12 the TAF alone group showed hematocrit levels higher than the other groups (*P* = 0.0317 compared to VEH group), except ARS + TAF group. The picture was similar on days 15 and 20, except that mice in the ARS only group had a recovery in hematocrit and showed no significant difference in relation to the TAF groups. **c** The profile of the platelet count decrease was similar to that of the hematocrit, decreasing in all groups on days 8 and 12 - the TAF alone group showed platelet counts higher than the other groups including the ARS + TAF group (*P* = 0.079 and 0.0456 compared to VEH on days 8 and 12, respectively). On day 20, only the groups VEH (*P* = 0.0159) and ARS + MEF (*P* = 0.0079) showed platelet counts significantly lower than the TAF group

We then ran a second experiment, this time using MEF again (alone or in combination with ARS) at 50 mg/kg but given by oral route to avoid toxicity. In this experiment, TAF alone or in combination with ARS was also assayed at the dose of 10 mg/kg (base). Dosing schemes for ARS (5 daily doses) and for MEF and TAF (3 alternate doses) were the same as in the previous experiment. MEF alone or in combination with ARS had no effect on parasite growth, compared to the group receiving vehicle only (Fig. 5). However, TAF alone or in combination with ARS showed potent inhibition of parasite growth after the second dose, leading to undetectable parasitemia (by microscopy) at day 9 post-infection. Parasitemia in these two groups remained undetectable by microscopy for the rest of the follow up (until day 30 post-infection). The effect on parasitemia also resulted in improved profiles for hematocrit and platelet counts in relation to all other groups (Fig. 6). Indeed, mice in the TAF-treated groups showed a milder decrease in these parameters, and recovered faster. There was little effect on body weight in any of the groups, except for a mild decrease at day 10, around the peak of parasitemia, in the groups, vehicle, ARS and MEF, but not TAF.

**Fig. 7:**
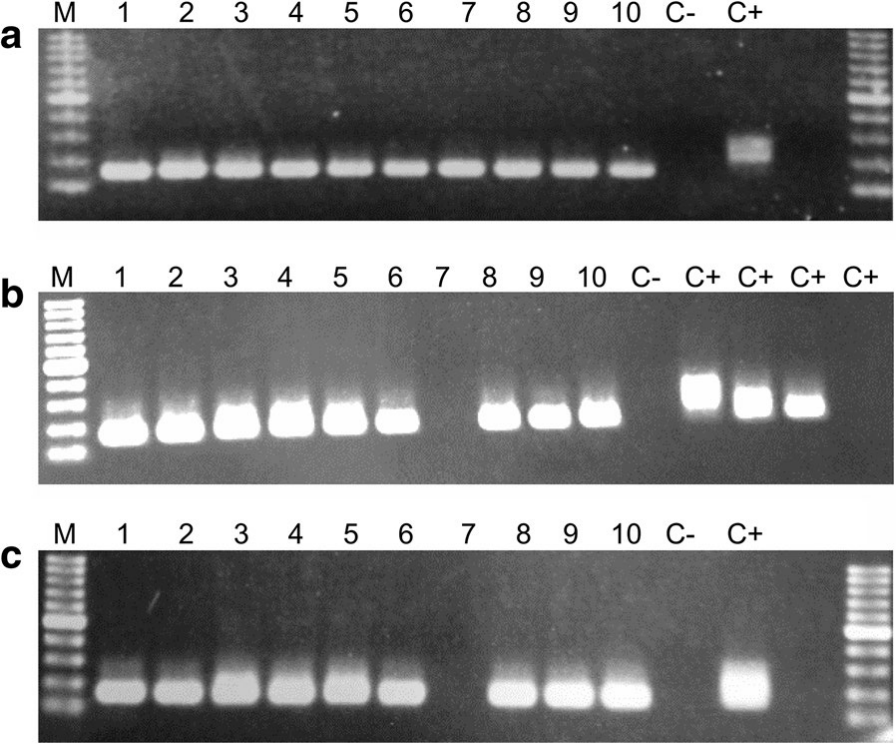
*Babesia microti*-specific PCR performed with DNAs from blood samples (10 μl blood diluted to a final volume of 100 μl in PBS, final dilution 1:10) of *B. microti*-infected mice treated with tafenoquine (samples 1–5) or artesunate + tafenoquine (samples 6–10) at days 15 (**a**), 20 (**b**) and 27 (**c**) post-infection. The last dose of tafenoquine was given on day 8 post-infection, and parasitemia was undetectable by microscopy from day 9 onwards. Positive control (C+) was DNA from blood of a *B. microti*-infected mouse with patent parasitemia by microscopy, and negative control (C-) was DNA from blood of a non-infected mouse. In the PCR on day 20 (**b**), the positive control sample (4% microscopy parasitemia) was run at four different dilutions: 1:10 (PCR+), 1:100 (PCR+), 1:1000 (PCR+) and 1;10,000 (PCR-) in PBS. *Abbreviation*: M, molecular weight marker

**Fig. 8:**
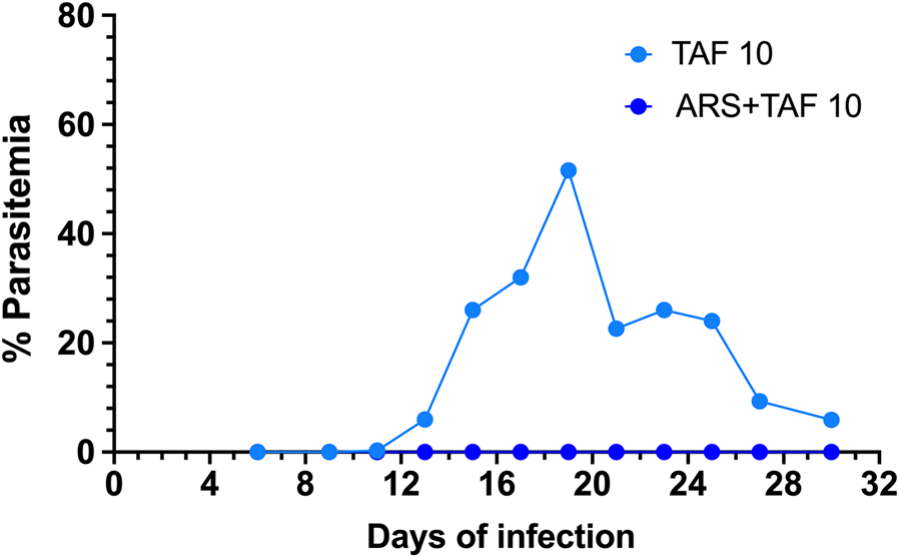
Curve of parasitemia (mean ± standard deviation) in BALB/c mice inoculated with blood of mice that had been previously infected with *Babesia microti* and treated with either tafenoquine (TAF) 10 mg/kg (base) or artesunate (ARS) 25 mg/kg + tafenoquine (TAF) 10 mg/kg (base). Blood (10 μl) was taken from the tail vein of each of the 5 infected and treated mice (total pooled = 50 μl) at day 30 of infection, when parasitemia was negative by microscopy but all but one mouse (group ARS + TAF) showed positive PCR for *Babesia microti*. The 50 μl of blood was administered intraperitoneally and parasitemia was checked by microscopy

However, despite clearing parasitemia by microscopy examination, all mice in the 2 TAF groups showed positivity for *B*. *microti* by PCR at day 15 post-infection (7 days after last TAF dose), and all but one (TAF plus ARS group) remained positive at days 20 and 27 post-infection (Fig. 7). Upon sub-inoculation, the mouse that received blood from the five mice treated with TAF alone developed parasitemia, whereas the mouse that received blood from the five mice treated with TAF plus ARS remained negative during the follow up (Fig. 8).

## Discussion

Here we attempted to use artesunate-based combinations as a potential strategy to improve efficacy of antimalarial drug treatment in *Babesia* infections. *In vitro*, combinations of artesunate with quinoline derivatives (the amino alcohol mefloquine and the 8-aminoquinolines tafenoquine and primaquine) resulted in better performances in terms of the IC_50_ than each drug alone. This effect was particularly strong for the artesunate-mefloquine combination, which resulted in a 20-fold increase in activity than mefloquine alone. However, unfortunately this marked increase in the *in vitro* activity was not observed in the *in vivo* conditions, even at the relatively high dose of 50 mg/kg of mefloquine and 25 mg/kg of artesunate. The reasons for the discrepancy between the *in vitro* and *in vivo* activities are not clear, but it may be related to pharmacokinetic or drug metabolism aspects. Whereas mefloquine is known to be a drug of slow clearance, remaining in the circulation for long periods after oral administration, artesunate is known to have the opposite behavior, with clearance time measured in hours [31, 32]. Therefore, contrary to the *in vitro* system where both drugs remain at fixed concentrations during the whole experiment, it is likely that *in vivo* adequate plasma concentrations are only achieved during a brief period of time after treatment and therefore synergy does not occur. Another possibility is that *B*. *microti* shows different susceptibility to the drug combination than *B*. *bovis*.

On the other hand, tafenoquine was shown to be highly effective in inhibiting *B*. *microti* growth *in vivo*, alone or in combination with artesunate. This inhibitory effect against *B*. *microti* has been shown before with higher doses (52 mg/kg and 16 mg/kg, base) in hamsters [11] and SCID mouse [12] models. In the present case, although parasitemia became rapidly undetectable by microscopy, mice remained positive by PCR even three weeks after they were negative by microscopy. These results suggest that tafenoquine, at the dose tested, was not potent enough to eliminate *Babesia* infections completely. But it is interesting that the blood of mice treated with the combination of tafenoquine plus artesunate was not infective when sub-inoculated in naive mice, indicating that the *B*. *microti* DNA detected by PCR in the blood of the donor mice in this group were from non-viable parasites. This result points to an increased efficacy of the combination tafenoquine plus artesunate compared to tafenoquine alone, however this conclusion requires additional evidence to be confirmed.

These results indicate that tafenoquine could be used in combination with the currently available drugs for animal babesiosis, such as diminazene aceturate, as a strategy to prevent development of resistance and also allowing the use of reduced doses of these drugs, which are commonly associated with a number of undesirable adverse effects at the conventional dosing schemes. One other possibility is to use tafenoquine as a starting point for medicinal chemistry modifications of its structure to identify compounds with increased efficacy and, eventually, lower costs. Indeed, this study and others [11, 12] show that 8-aminoquinolines such as tafenoquine itself, primaquine and 4-methylprimaquine show good efficacy *in vitro* and *in vivo* against *Babesia*, and therefore derivatives with better activity profiles against *Babesia* may be discovered with this approach.

## Conclusions

The present study evaluated ACTs against babesiosis and found that the combination of artesunate-tafenoquine is a potential novel chemotherapeutic regimen. Our findings also suggest that tafenoquine and potentially other 8-aminoquinolines are a promising class of drugs with solid potential as new chemotherapeutics for babesiosis.

## Data Availability

Data supporting the conclusions of this article are included within the article.

## Abbreviations

ACT: artemisinin-based combination therapy
ARS: artesunate
LUM: lumefantrine
MB: methylene blue
MEF: mefloquine
PRI: primaquine
TAF: tafenoquine
VEH: vehicle
DDW: double distilled water
DMSO: dimethylsulphoxide
DNA: deoxyribonucleic acid
rRNA: ribosomal ribonucleic acid
IC_50_: half maximal inhibitory concentration
IP: intraperitoneal
PCR: polymerase chain reaction
PEG: polyethyleneglycol
PO: *per os*
RBC: red blood cells
iRBC: parasitized red blood cells
uRBC: uninfected red blood cells
SCID: severe combined immunodeficiency
